# Reference values for bioelectrical impedance–derived phase angle: a cross-sectional study of 5,049 healthy individuals from Colombia

**DOI:** 10.1038/s41430-026-01744-z

**Published:** 2026-04-18

**Authors:** Frank Carrera-Gil, Robinson Ramírez-Vélez, Gildardo Uribe-Gil

**Affiliations:** 1 Departamento de Alimentación y Nutrición, Facultad de Ciencias de la Salud, Pontificia Universidad Javeriana Seccional Cali, Cali, Colombia; 2 Navarrabiomed, Hospital Universitario de Navarra (HUN), Navarra Institute for Health Research (IdiSNA), Universidad Pública de Navarra (UPNA), Pamplona, Spain; 3 CIBER of Frailty and Healthy Aging (CIBERFES), Instituto de Salud Carlos III, Madrid, Spain; 4 Facultad de Ciencias de la Educación, Unidad Central del Valle del Cauca (UCEVA), Tuluá, Colombia; 5 Programa de Nutrición y Dietética, Facultad de Ciencias de la Salud, Fundación Universitaria María Cano, Medellín, Colombia; 6 Present Address: Connected Nutrition Ventures, Medellín, Colombia

**Keywords:** Biomarkers, Nutrition

## Abstract

**Background and aims:**

Phase angle (PhA), derived from bioelectrical impedance analysis (BIA), is a noninvasive indicator of cellular integrity and body cell mass. PhA varies with age, sex, and population background; however, reference data from Latin American populations remain limited. This study therefore aimed to establish age- and sex-specific PhA reference values and percentile curves in a healthy Colombian population.

**Methods:**

This cross-sectional study enrolled 5,049 individuals (3,113 females) aged 10–94 years. PhA was calculated directly from body resistance and reactance obtained from BIA. Smoothed centile curves and tables for the 3rd, 10th, 25th, 50th, 75th, 90th, and 97th percentiles were calculated using the Lambda-Mu-Sigma (LMS) method to develop sex- and age-specific norms.

**Results:**

PhA followed a non-linear pattern with age, increasing during adolescence and early adulthood, peaking around midlife (40–49 years), and declining thereafter. Males consistently exhibited higher PhA values than females across all age groups. Sex-specific percentile curves (P3–P97) were generated, illustrating the distribution of the PhA by age group and providing normative reference values for clinical and research applications.

**Conclusion:**

This study provides the first age- and sex-specific phase angle reference values for a healthy Colombian population, addressing the lack of normative standards in Latin America. These population-specific curves offer clinically relevant benchmarks that may improve the assessment of cellular health and nutritional status in diverse settings.

## Introduction

In recent years, phase angle (PhA), measured by bioelectrical impedance analysis (BIA), has emerged as a useful indicator of cellular integrity and overall health [1]. PhA reflects the relationship between cellular resistance and reactance [2] and is considered a raw parameter, independent of predictive equations or anthropometric variables such as body weight or height [1, 3]. This characteristic supports its use in clinical contexts where conventional measurements are limited.

Substantial evidence indicates that PhA is associated with clinically relevant outcomes, including mortality, postoperative complications, length of hospital stay, and disease prognosis [4–12]. PhA has also been linked to nutritional status and functional capacity and may detect early cellular alterations before changes in body weight or biochemical markers become evident [13–18].

The clinical interpretation of PhA relies on reference values derived from healthy populations. However, PhA varies according to age, sex, and population background, underscoring the need for population-specific reference standards [19]. Most available reference data have been generated in European and North American populations [20–22], which may not adequately represent other geographic or demographic contexts. Given the heterogeneous composition of the Colombian population, characterized by admixture among individuals of European, Indigenous, and African ancestry [23], the use of external reference values may be inappropriate.

Therefore, the aim of this study was to develop age- and sex-specific percentile reference curves and values for PhA in a large sample of healthy Colombian individuals.

## Materials and methods

### Study design and participants

This cross-sectional study was conducted across the listed Colombian regions, from June 2019 to January 2025. A total of 5049 individuals (3,113 females) aged 10 to 94 years were enrolled. The study included healthy participants from four cities in Colombia—Bogotá (*n* = 1,200), Cartagena (*n* = 800), Medellín (*n* = 1,200), and Pereira (*n* = 649)—and from three small towns—Zaragoza (*n* = 250), Remedios (*n* = 350), and Marmato (*n* = 600). Recruitment and assessments were conducted locally in each city by trained personnel following standardized procedures. Individuals were eligible if they provided informed consent before participation. For minors under 18 years of age, written informed consent was obtained from a parent or legal guardian, and assent was sought from the participant whenever possible, in accordance with the ethical guidelines for research involving minors. The exclusion criteria included pregnancy, self-reported acute or chronic disease, the presence of implanted electronic devices (e.g., pacemakers) or metallic prosthetics, limb amputation, and menstruation at the time of assessment.

### Data sources

Trained evaluators followed the standards of the International Society for the Advancement of Kinanthropometry (ISAK) for anthropometric measurements [24]. Body weight was measured using a digital scale (Seca 874, Hamburg, Germany) with participants wearing light clothing and no shoes, and height was measured using a stadiometer (Seca 213, Hamburg, Germany) to the nearest 0.1 cm. Body mass index (BMI) was calculated as weight in kilograms divided by height in meters squared.

Body composition was assessed in the seated position using a hand-to-foot, tetrapolar, multifrequency bioelectrical impedance analyzer (BiodyXpert ZM II, Aminogram SAS, France). This Class IIa European medical device has been validated against dual-energy X-ray absorptiometry (DXA) in healthy young adults [25], provides reproducible estimates of appendicular skeletal muscle mass (ASM, 0.32 ± 0.85 kg) in older hospitalized patients [26], and is sensitive to PhA changes associated with systemic inflammation in overweight young women [27]. In seated measurements, analyzers from the same device family have shown acceptable agreement with DXA for fat mass (FM) and fat-free mass (FFM) in healthy male adults, with mean deviations of 1–2%, strong correlations (r = 0.78–0.88), and high intra-rater reliability (intraclass correlation coefficients: 0.81–1.00) [28].

BIA measurements were performed after an 8-hour fast, following standard conditions to minimize hydration-related variability: participants refrained from exercise, alcohol, caffeine, and diuretics for at least 24 h and emptied their bladder before assessment. Each participant was seated barefoot on the front edge of a non-metallic chair, with the left knee bent at 90° and the left hand resting on the left knee. The right foot was positioned slightly backward without touching the chair leg, keeping the heel and toes at a 90° angle, while maintaining an upright posture. Prior to measurement, tactile electrodes were moistened to ensure optimal conductivity. Hand electrodes were placed over three fingers with the thumb on the switch electrode, and foot electrodes were positioned on both sides of the malleoli.

The BIA device measured R and Xc using an alternating current of 50 kHz and 800 µA, and computed PhA at the same frequency as arctangent (Xc/R) × (180/π). Using these impedance measurements, the analyzer estimated ASM and FFM through the predictive equations of Kyle et al. [29, 30], which show high validity compared with DXA (ASM: r = 0.95, SEE = 1.12 kg; FFM: r = 0.986, SEE = 1.72 kg). ASM was adjusted for height to obtain the appendicular skeletal muscle mass index (ASMI; ASM/height², kg/m²). FM was expressed as a percentage (FM%) and calculated by subtracting FFM from total body weight; FFM values were not reported because they were used solely to derive FM.

### Data analysis

We used SPSS version 26.0 software for Windows (SPSS, Chicago, Illinois, USA) for all analyses except for the Lambda-Mu-Sigma (LMS) calculations. The normality of the selected variables was verified using histograms and Q-Q plots. Outliers in PhA were excluded when they fell outside the boxplot whiskers, defined as values exceeding 1.5 times the interquartile range (IQR). Descriptive statistics are presented as the median (IQR) for continuous variables and as numbers and frequencies for categorical variables.

Spearman’s rank correlation coefficients were calculated for age, BMI, FM%, and ASMI to assess the strength and direction of their relationships with PhA. A *p* value < 0.05 was considered statistically significant.

A generalized additive model (GAM) was fitted to explore the association between age and PhA, allowing for non-linear effects using penalized cubic splines. Sex was included as a grouping variable to assess sex-specific trajectories across the lifespan. The shaded area in the plot represents the 95% confidence interval for the smoothed curve.

To develop age- and sex-specific reference curves for PhA, the LMS method was applied. This method assumes that the outcome variable follows a normal distribution after a Box–Cox power transformation. LMS calculations were performed using the LMS Chart Maker Pro Version 2.54 (Medical Research Council, London, UK), and smoothed curves for each age were obtained via penalized maximum likelihood with the following abbreviations: M (median), L (Box–Cox transformation), and S (coefficient of variation) [31]. The model providing the best balance between fit and complexity (i.e., degree of smoothing) was selected using the Akaike information criterion. The appropriate number of degrees of freedom was selected based on deviance, Q-tests, and worm plots, following the suggestions of Royston and Wright [32].

The 3rd, 10th, 25th, 50th, 75th, 90th, and 97th percentiles were fitted separately for males and females within each decade-of-life age group (10–19, 20–29, 30–39, 40–49, 50–59, 60–69, and ≥70 years), as done in previous studies [20–22]. Norms are tabulated as percentile values and visualized as smoothed percentile curves.

## Results

Table 1 shows the general characteristics of the participants. The median age was 24.7 years, and the median BMI was 24.3 kg/m², with no significant differences in age between sexes. Males had significantly higher height, body weight, and ASMI values than females. In contrast, females had significantly higher BMI and a greater FM% (*p* < 0.001 for all comparisons).

**Table 1:** Characteristic of participants.

Variables	Full Sample	Male	Female	*p* value
	(n = 5,049)	(n = 1,936)	(n = 3,113)	
Age (y)	24.7 (19.3–45.7)	23.9 (19.7–45.7)	25.6 (19.6–45.8)	0.868
Height (cm)	162 (156–170)	172 (166.1–177)	152.2 (153.2–162.8)	<0.001
Weight (kg)	64.9 (56.6–75)	70.4 (62–80.1)	61.8 (54.4–70)	<0.001
BMI (kg/m^2^)	24.3 (21.6–27.6)	24.2 (21.4–26.9)	24.5 (21.8–28.1)	<0.001
FM (%)	20.7 (27.4–33.4)	13.5 (19.0–23.9)	27.1 (31.5–35.8)	<0.001
ASMI (kg/m^2^)	7.0 (6.2–7.9)	7.9 (7.3–8.5)	6.4 (5.9–7.1)	<0.001
Phase Angle (°)	6.8 (6.3–7.4)	7.4 (6.9–7.8)	6.5 (6.1–7.0)	<0.001

Data expressed as median (interquartile range). *y* years, *BMI* Body mass index, *FM* Fat mass, *ASMI* Appendicular skeletal muscle mass index.

Spearman correlations were used to examine the relationships between PhA, body composition, and age. A weak positive association was observed with BMI (rho = 0.226, *p* = 0.001), while weak negative associations were found with FM% (rho = –0.211, *p* = 0.001) and age (rho = –0.122, *p* = 0.001). In contrast, a strong positive association was detected with ASMI (rho = 0.652, *p* = 0.001).

**Fig. 1:**
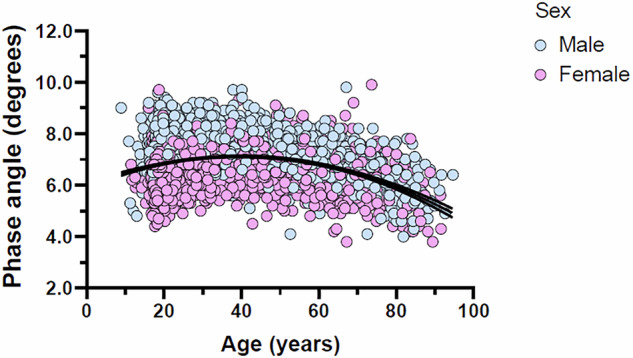
Scatter plot with fitted curve for phase angle according to sex and age group. Scatterplot illustrating individual values of phase angle (y-axis) as a function of age in years (x-axis) for male (blue) and female (pink) participants. A non-linear (quadratic) regression line (black) with 95% confidence intervals is overlaid to depict the overall trend. Phase angle shows a modest increase during early adulthood, peaking around midlife, followed by a progressive decline with advancing age. Sex differences are apparent across the age span, with males generally exhibiting higher phase angle values than females.

Figure 1 shows the relationship between age and PhA in a large cross-sectional sample, stratified by sex. A curvilinear (quadratic) trend was observed, with PhA values increasing during early adulthood, reaching a peak in midlife, and subsequently declining with advancing age. Males exhibited consistently higher PhA values than females across the entire age span. The fitted regression line and its 95% confidence intervals depict the non-linear trajectory of PhA across age and emphasize the differences between sexes.

**Fig. 2:**
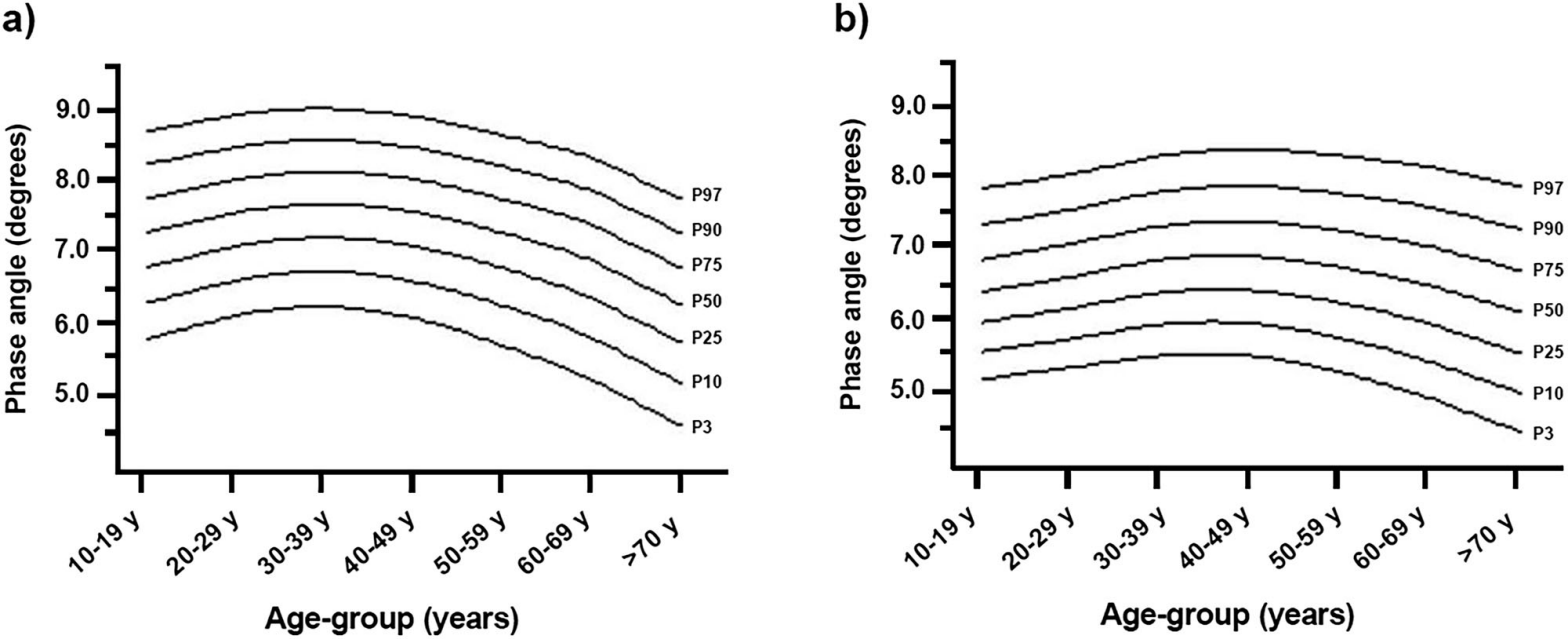
Smoothed percentile curves (P3 to P97) for phase angle according to sex and age group. Male (Panel **a**) and female (Panel **b**). P, percentile.

Figure 2 presents age-specific percentile curves (P3 to P97) of PhA by sex. Among males (Fig. 2a), PhA increased from adolescence to midlife across all percentiles, with the median (P50) rising from approximately 7.3° in the 10–19-year group to 7.6° in the 40–49-year group, followed by a steady decline thereafter. A similar pattern was observed in females (Fig. 2b), although the median PhA values were consistently lower than in males across all age groups. These percentile curves provide normative reference values and illustrate the expected distribution of PhA across age and sex groups, as detailed in Table 2.

**Table 2:** Reference values (percentiles) for phase angles according to sex and age group.

	n	L	S	P3	P10	P25	P50	P75	P90	P97
Male										
10–19 y	533	1.3	0.1	5.8	6.3	6.8	7.3	7.8	8.2	8.7
20–29 y	618	1.3	0.1	6.2	6.6	7.1	7.6	8.0	8.5	8.9
30–39 y	190	1.4	0.1	6.3	6.8	7.2	7.7	8.1	8.6	9.0
40–49 y	193	1.6	0.1	6.1	6.6	7.1	7.6	8.0	8.5	8.9
50–59 y	147	1.7	0.1	5.7	6.3	6.8	7.3	7.7	8.2	8.6
60–69 y	102	1.6	0.1	5.3	5.8	6.4	6.9	7.4	7.9	8.3
>70 y	153	1.6	0.1	4.6	5.2	5.8	6.3	6.8	7.3	7.7
Female										
10–19 y	868	0.1	0.1	5.2	5.6	5.9	6.3	6.8	7.2	7.7
20–29 y	891	0.4	0.1	5.4	5.7	6.1	6.5	7.0	7.4	7.9
30–39 y	352	0.6	0.1	5.5	5.9	6.3	6.8	7.2	7.7	8.1
40–49 y	365	0.6	0.1	5.5	5.9	6.4	6.8	7.3	7.7	8.2
50–59 y	287	0.7	0.1	5.3	5.7	6.2	6.7	7.1	7.6	8.1
60–69 y	223	0.8	0.1	4.9	5.4	5.9	6.4	6.9	7.4	8.0
>70 y	127	0.8	0.1	4.5	5.0	5.5	6.1	6.6	7.2	7.7

P, percentile; y, years; L, Box–Cox transformation used to normalize the distribution of values; S, coefficient of variation indicating the relative spread of the data around the median.

## Discussion

This study established reference values and percentile curves for PhA in healthy individuals from adolescence to late adulthood in a Colombian population, providing new reference data from an underrepresented region and contributing to the international evidence on age- and sex-related variation in PhA. Consistent with previous findings, PhA values were higher in males than females, likely reflecting their greater skeletal muscle mass. Our correlation analysis further confirmed that PhA increases in parallel with skeletal muscle mass, supporting the concept that PhA reflects the volume of this tissue.

In both sexes, PhA increased from adolescence to early adulthood and declined after age 50, an age-related pattern consistent with previous findings in Italian adults [22], although the decline occurred slightly later in our population. Notably, when matched by age and sex, Colombian participants exhibited higher median PhA values than those reported in Germany [20], the United States (U.S) [21], Italy [22], and in a meta-analysis of mean PhA values from 46 studies [33] (Table 3). For example, in the 20–29-year age group, PhA was 7.6° in males and 6.5° in females, compared with 7.0° and 6.1° in Germans—mean differences of 0.6° and 0.4°, respectively.

**Table 3:** Comparison of phase angle values (50th percentile or mean) from large studies.

Age (years)	Present study (p 50th)		Germany (Mean)		U.S Hispanic/black (Mean)		U.S White/other (Mean)		Italy (p 50th)		Meta-analysis (Mean)	
	♂	♀	♂	♀	♂	♀	♂	♀	♂	♀	♂	♀
<20	7.3	6.3	7.1	6.1	6.4	5.4	6.4	5.3	7.1	6.2	–	–
20–29	7.6	6.5	7.0	6.1	6.5	5.5	6.3	5.5	7.3	6.2	6.9	6.1
30–39	7.7	6.8	6.9	6.2	6.7	5.6	6.3	5.4	7.4	6.3	7.2	6.2
40–49	7.6	6.8	6.7	6.1	6.1	5.4	6.1	5.1	7.2	6.2	7.0	6.3
50–59	7.3	6.7	6.4	5.9	–	–	–	–	6.9	5.9	6.5	5.9
60–69	6.9	6.4	6.0	5.6	–	–	–	–	6.2	5.8	6.5	5.6
> 70	6.3	6.1	5.4	5.3	–	–	–	–	–	–	–	–

Germany [20]; United States (U.S) [21]; Italy [22]; Meta-analysis [33]. P, percentile. Male ♂, Female ♀.

These differences may be partly attributable to technical and methodological variations among the BIA devices used. Although all studies employed tetrapolar, hand-to-foot analyzers operating at 50 kHz, they differed in measurement posture and device models. In our study, participants were assessed seated using a multifrequency analyzer (BiodyXpert ZM II, Aminogram SAS, France), whereas European and U.S. studies measured supine using different devices: the German dataset used the single-frequency BIA 2000-S (Data Input, Frankfurt, Germany); the Italian study employed the BIA 101 or BIVA PRO (Akern, Italy); and the U.S. study used the multifrequency Hydra 4200 (Xitron Technologies, San Diego, CA).

Posture affects body fluid distribution in bioelectrical impedance measurements [34], but the evidence regarding its actual impact on PhA values is inconsistent. Więch et al. reported higher PhA values in the sitting position compared with standing, and similar values between sitting and supine when using the BIA 101 analyzer. In their study, men showed mean PhA values of 7.23° when sitting, 6.27° when standing, and 7.03° in supine, with significant differences between sitting and standing [35]. This pattern is often explained by posture-related shifts in body fluids: standing promotes extracellular fluid pooling in the lower extremities, increasing resistance and tending to lower PhA, whereas sitting and supine positions support more uniform fluid distribution [34]. However, empirical findings do not consistently follow this physiological model, indicating that posture alone cannot fully explain the observed variability. In fact, Espasa-Labrador et al. reported the opposite pattern: when comparing the BiodyXpert ZM III in a seated position with the BIA 101 measured in supine, the BiodyXpert significantly underestimated PhA by approximately 0.7 units (9% lower) [28], highlighting that discrepancies between devices likely arise from methodological and technical differences rather than posture alone.

Additional technical factors, such as electrode characteristics, can also influence PhA: in measurements performed supine with the Nutriguard-M analyzer (Data Input, Germany), electrodes with smaller conductive gel areas produced lower values compared with larger contact surfaces (5.7 ± 0.9° vs. 6.5 ± 0.6°) [36].

Beyond technical considerations, population-specific factors contribute to PhA variability. Jensen et al. demonstrated that, using identical devices and measurement conditions (seca mBCA 515, 50 kHz, hand-to-foot standing), Mexicans exhibited higher PhA than Germans and Japanese across all age groups (18–74 years), despite Germans having greater height, body weight, fat-free mass, and skeletal muscle mass [37]. These findings suggest that PhA is influenced by determinants beyond body composition, including physiological and lifestyle factors.

Dietary patterns, in particular, may modulate PhA. Emerging evidence shows that PhA is negatively associated with the intake of ultra-processed foods (UPFs) [38] and increases proportionally with greater adherence to healthy diets, such as the Mediterranean diet [39]. In Colombia, UPFs account for approximately 15% of total energy intake, compared with 40–60% reported in several high-income countries [40, 41]. Diets high in UPFs are associated with systemic inflammation [42], which may impair cell membrane integrity and lower PhA [4]. Conversely, lower UPF consumption in Colombians may support better cellular function, contributing to the higher PhA observed.

Taken together, these findings underscore the need to interpret PhA using population- and device-specific reference values whenever possible.

Finally, although most studies define low PhA as values below the 5th percentile adjusted for age and sex [6, 20, 21], no universal cutoff has been established, and it remains uncertain whether the 5th percentile—or another threshold—best identifies individuals at increased clinical risk. Therefore, PhA should be interpreted within its clinical context and alongside other biomedical data.

Evidence showing that resistance exercise [16] and healthy dietary patterns [39] can increase PhA supports the clinical relevance of identifying individuals with low values. However, prospective studies are still required to evaluate the prognostic value of PhA using these new reference values and to determine the optimal cutoff for detecting low PhA in the Colombian population.

### Limitations and strengths

A key strength of this study is its large sample size and broad regional representation, which supported the development of age- and sex-specific PhA reference curves for a population largely underrepresented in international research. The use of the LMS method and rigorous statistical criteria ensured stable and accurate percentile estimates. By providing Colombian-specific normative values, this study fills an important gap and offers context-appropriate reference data for populations with heterogeneous population backgrounds, where physiological, environmental, and lifestyle factors may influence PhA differently from those in high-income countries.

This study also has several limitations. First, defining age intervals based on population deciles resulted in a broad 10–20-year category. Although this ensured sufficient sample size for stable LMS estimation, it groups individuals undergoing distinct developmental and physiological stages, potentially obscuring meaningful within-group variability. Second, despite including participants from multiple regions, the sample may not fully capture Colombia’s demographic diversity; rural, low-socioeconomic, and ethnic minority groups may be underrepresented, and the lack of detailed ethnic characterization limits interpretation of potential population-related differences in PhA. Finally, the cross-sectional design introduces the possibility of cohort effects, meaning that differences between age groups may reflect generational exposures rather than physiological aging. Longitudinal studies are needed to determine whether the percentile curves generated here accurately represent individual trajectories over time.

## Conclusion

This study provides the first age- and sex-specific reference values and percentile curves for phase angle in a large sample of healthy Colombians, demonstrating clear age-related patterns and differences compared with European and North American populations. These findings underscore the need for population-specific normative data rather than reliance on reference values derived from other geographic or demographic contexts. The resulting curves offer clinicians and researchers a practical tool for interpreting PhA in this population and may enhance the identification of individuals who could benefit from nutritional or functional interventions.

Future studies should assess the prognostic value of these reference ranges, determine the most appropriate cutoff for low PhA in Colombians, and examine how factors such as ethnic composition, socioeconomic conditions, and urban–rural differences influence PhA distributions. Longitudinal research is also needed to evaluate within-individual changes over time and determine whether the percentile curves reflect clinically meaningful trajectories across demographic groups.

## Data Availability

The datasets generated and/or analyzed during the current study are available from the corresponding author upon reasonable request.
